# Developing and Characterizing the Tumor-Targeting Efficiency of an Anti-EphA2-CD11b Bispecific Antibody

**DOI:** 10.1021/acs.bioconjchem.5c00070

**Published:** 2025-05-28

**Authors:** Peggy A. Birikorang, Dominic M. Menendez, Robert Edinger, Gary Kohanbash, W. Barry Edwards

**Affiliations:** † Department of Biochemistry, 14716 University of Missouri-Columbia, 503 S College Avenue, Columbia, Missouri 65211, United States; ‡ Molecular Imaging and Theranostics Center, University of Missouri-Columbia, 1514 Research Park Drive, Columbia, Missouri 65203, United States; § Department of Radiation Oncology, University of Pittsburgh, 200 Lothrop Street, Pittsburgh, Pennsylvania 15213, United States; ∥ Department of Neurological Surgery, University of Pittsburgh, 503 45th Street, Pittsburgh, Pennsylvania 15201, United States; ⊥ Department of Chemistry, University of Missouri-Columbia, 601 S College Avenue, Columbia, Missouri 65211, United States

## Abstract

Targeting molecules, such as antibodies and peptides, play a key role in the precise delivery of cytotoxic payloads to tumor sites by binding to specific tumor-associated antigens or other proteins within the tumor microenvironment. This investigation evaluates the potential therapeutic application of a bispecific antibody (BsAb), which simultaneously targets EphA2, a tumor-associated antigen, and CD11b, a protein expressed by tumor-associated macrophages and myeloid-derived suppressor cells (TAMCs). Recombinantly produced anti-EphA2-CD11b-BsAb was conjugated to a bifunctional chelator, NOTA-SCN, and then radiolabeled with copper-64 (^64^Cu). The [^64^Cu]­Cu-NOTA-anti-EphA2-CD11b-BsAb radioimmunoconjugate was subsequently administered to HT1080-fibrosarcoma-bearing nude mice via tail vein injection. Positron Emission Tomography (PET) and ex vivo biodistribution analyses were performed to determine tumor uptake and pharmacokinetic localization. At 4, 24, and 48 h postinjection (p.i.), the percent injected dose per gram (%ID/g) of [^64^Cu]­Cu-NOTA-anti-EphA2-CD11b-BsAb in HT1080 xenografts were 5.35 ± 2.24, 4.44 ± 1.90, and 4.10 ± 0.60, respectively. There was high uptake in the liver as well as in CD11b-expressing organs, including the spleen, bone marrow, and lung. Binding in these CD11b-rich organs was significantly reduced by coadministering the dose with nonradiolabeled anti-CD11b-IgG and anti-EphA2-CD11b-BsAb, with a concurrent increase in tumor uptake compared to nonblocked mice (8.39 ± 1.37%ID/g for blocked and 4.44 ± 1.90%ID/g for nonblocked at 24 h p.i., *p* = 0.0175). Further optimization studies showed that at lower molar activity (3.7 MBq/nmol, 100 μCi/nmol), there were significantly higher tumor accumulations and reduced uptake in CD11b-expressing organs compared to higher molar activity (22.2 MBq/nmol, 600 μCi/nmol). Anti-EphA2-CD11b-BsAb is a functional targeting molecule and would require optimization through molar activity or blocking with nonradiolabeled antibody to maximize tumor targeting.

## Graphical abstract

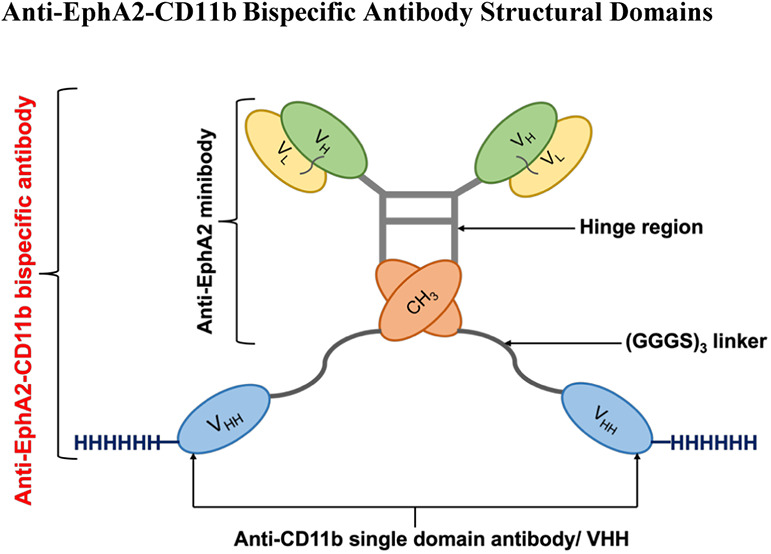

## Introduction

The aim of this study is to assess and optimize the tumor-targeting ability and biodistribution of a bispecific antibody (BsAb) targeting both EphA2, a tumor cell marker, and CD11b, a cell surface marker expressed on tumor-associated macrophages and myeloid-derived suppressor cells (TAMCs), to gauge its potential as a therapeutic targeting molecule. The presence and abundance of proteins that are upregulated as a function of disease in tumors have been crucial for the development of cancer therapies. Antigen-targeted therapies, including immunotherapies, antibody-drug conjugates, and targeted radionuclide therapies, aim to bind to specific proteins for precise delivery of cytotoxins, immune cells, radionuclides, etc., to the tumor site, minimizing adverse effects on normal tissues.^1–3^ This targeted approach gives antigen-targeted therapies a putative edge over chemotherapy and external-beam radiation in cancer treatment in terms of specificity and reduced toxicity.^1,4^ Most of these targets consist of proteins and markers overexpressed on the tumor cell surface with minimal to no expression on normal cells in healthy tissues,^5,6^ as exemplified by EphA2.

EphA2, a tyrosine kinase cell surface receptor, is abundantly expressed across various cancer types, including breast cancer, glioblastoma, melanoma, and lung cancer.^7,8^ Mounting evidence suggests that EphA2 overexpression promotes tumor growth and metastasis by augmenting cell proliferation, migration, invasion, and immunosuppression.^9–11^ Given its limited expression in healthy tissues, numerous studies have targeted EphA2 for various cancer therapeutic purposes, such as EphA2-based peptide vaccines and EphA2 CAR-T cell therapy,^12–15^ which are currently undergoing clinical trials in human patients.

The tumor microenvironment contains TAMCs, which contribute to an immunosuppressive environment.^16,17^ Due to the heavy infiltration of TAMCs in the tumor microenvironment, CD11b, a cell surface protein expressed by these cells, presents an ideal target for the selective delivery of cytotoxins for TAMC depletion. Putatively, a reduction in TAMCs could result in increased immune checkpoint immunotherapeutic efficacy. Numerous studies have specifically targeted CD11b for cancer therapeutics^18–23^ as well as noninvasive diagnostic imaging of tumors.^24,25^

While many antigen-targeted therapeutic agents typically focus solely on either tumor cell antigens or noncancer cell markers within the tumor microenvironment, targeting both components simultaneously could potentially enhance overall therapeutic efficacy. As an initial step in exploring this concept, we developed and assessed the in vivo tumor-targeting and pharmacokinetic localization of a dual-targeting molecule: an anti-EphA2-CD11b-BsAb. This BsAb was engineered to bind to both the tumor-associated antigen EphA2 and the tumor microenvironment-rich protein CD11b, achieved by linking the sequence of an anti-CD11b single-domain antibody (VHH) to the C-terminal of an anti-EphA2 minibody via a flexible glycine–serine-rich linker.

To commence our investigation, we produced the anti-EphA2-CD11b-BsAb through recombinant protein expression in mammalian cells and then assessed its purity, molecular weight, and binding affinities to both EphA2 and CD11b antigens. Subsequently, the BsAb was conjugated to NOTA and radiolabeled with the positron-emitting radionuclide copper-64 (^64^Cu). The resultant radioimmunoconjugate was administered to HT1080-fibrosarcoma-bearing mice for in vivo positron emission tomography (PET) and ex vivo biodistribution. Preliminary findings revealed high and rapid in vivo uptake of [^64^Cu]­Cu-NOTA-anti-EphA2-CD11b-BsAb in HT1080 xenografts, as well as high uptake in CD11b-rich organs such as the spleen, bone marrow, and lung.^26–28^ This may pose a significant limitation to the potential therapeutic application of the BsAb, as the goal of targeted therapies is to minimize uptake and thus adverse effects on normal tissues. To reduce uptake in these CD11b-rich organs, the initial dose of [^64^Cu]­Cu-NOTA-anti-EphA2-CD11b-BsAb was coadministered with nonradiolabeled anti-EphA2-CD11b-BsAb and anti-CD11b antibody (IgG) to occupy the antigen sink and inhibit the uptake of the radiolabeled conjugate. Compared to the nonblocked group, blocked mice had [^64^Cu]­Cu-NOTA-anti-EphA2-CD11b-BsAb uptake significantly reduced, with a concurrent increase in tumor uptake. Altering molar activities by varying the ratio or amount of radioactivity to equal amounts of BsAb further showed enhanced tumor uptake at lower molar activities in comparison to higher molar activities.

These findings, therefore, suggest that, with suitable optimization to minimize uptake in nontarget tissues, anti-EphA2-CD11b-BsAb emerges as a promising targeting molecule for delivering various cytotoxic agents to both the tumor and its microenvironment for therapeutic purposes.

## Results

### Purity, Stability, and Molecular Weight of Recombinantly Produced Anti-EphA2-CD11b-BsAb

Anti-EphA2-CD11b-BsAb was designed from two antibody fragments: an anti-EphA2 minibody with variable heavy (V_H_), variable light (V_L_),^29^ and constant heavy (CH_3_) domains linked to an anti-CD11b VHH.^25^ A cartoon of the various structural domains is shown below (Figure 1A). Following sequence design, recombinant production, and purification, SDS-PAGE and SEC-HPLC were performed to obtain purity, stability, and molecular weight. The molecular weight of anti-EphA2-CD11b-BsAb, 116 693 Da, calculated from the amino acid sequence (ExPASy ProtParam), was consistent with the molecular weights obtained from SDS-PAGE and SEC-HPLC, in reference to the molecular weight standards (Figure 1B,C, respectively). Percent purity obtained via SEC-HPLC was 97%, with no apparent aggregation.

**Figure 1:**
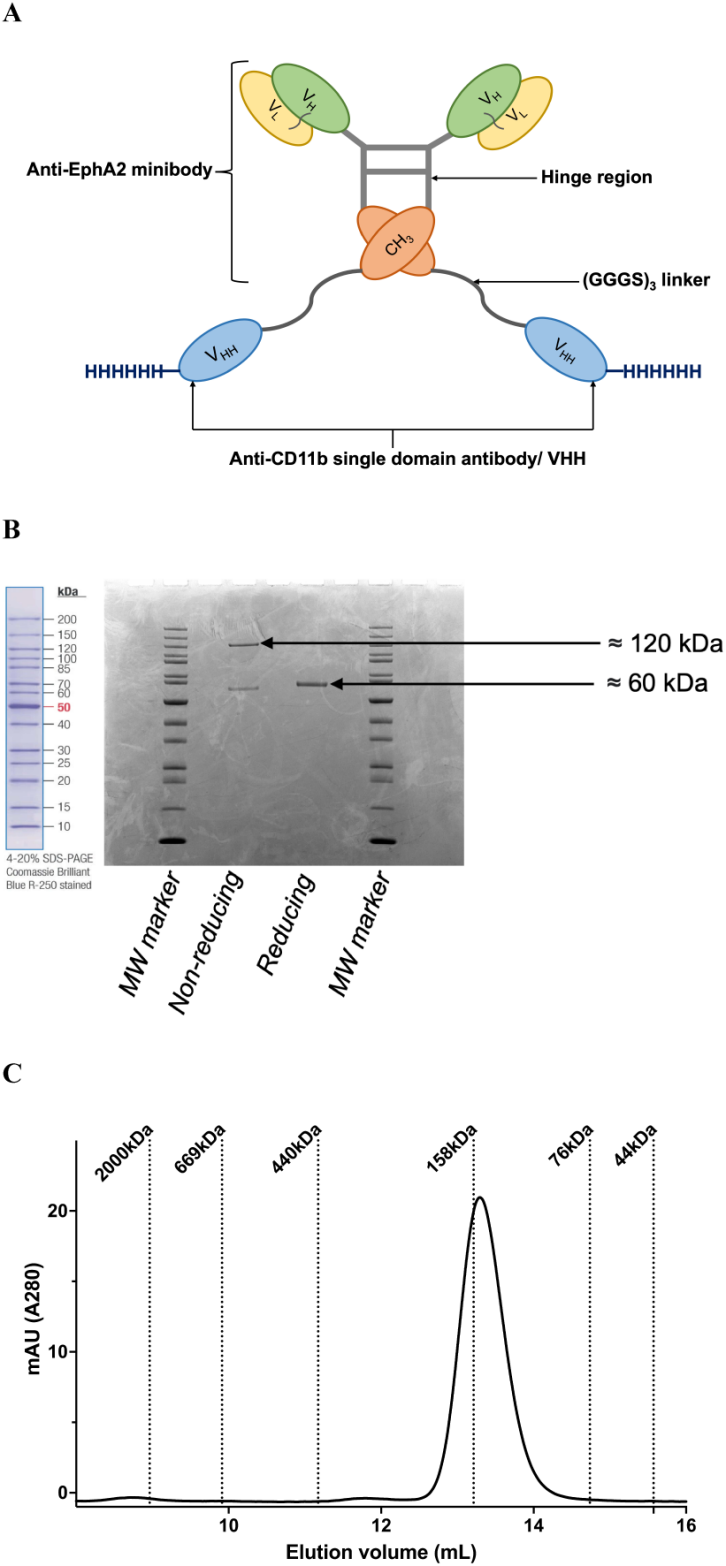
Characterization of the molecular weight and purity of recombinant anti-EphA2-CD11b-BsAb. (A) Cartoon depicting the anti-EphA2-CD11b-BsAb structure, comprising an anti-EphA2 minibody and an anti-CD11b-VHH fused via a flexible glycine-serine linker, with a histidine tag at the C-terminus. (B) SDS-PAGE image of anti-EphA2-CD11b-BsAb performed under nonreducing and reducing conditions, with resultant molecular weights of about 120 kDa (homodimer) and 60 kDa (monomer), respectively. (C) SEC-HPLC chromatogram of anti-EphA2-CD11b-BsAb with reference to molecular weight standards

### Binding Affinity of Anti-EphA2-CD11b-BsAb to Target Antigens EphA2 and CD11b

Saturation binding assays performed using immobilized human EphA2 antigen and CD11b-expressing Raw 264.7 cells showed that anti-EphA2-CD11b-BsAb has high binding affinities for both molecular targets, EphA2 and CD11b. *K_D_* values obtained were 0.54 ± 0.19 nM for EphA2 and 1.19 ± 0.44 nM for CD11b (Figure 2AB, respectively). Binding assays were also conducted for NOTA-anti-EphA2-CD11b-BsAb to confirm that the conjugation reaction did not disrupt the affinity of binding to the target antigens. NOTA-anti-EphA2-CD11b-BsAb maintained high binding affinities, with *K_D_* values of 1.47 ± 0.46 nM and 1.59 ± 0.58 nM for EphA2 and CD11b, respectively (Figure 2C,D) (*n* = 3).

**Figure 2:**
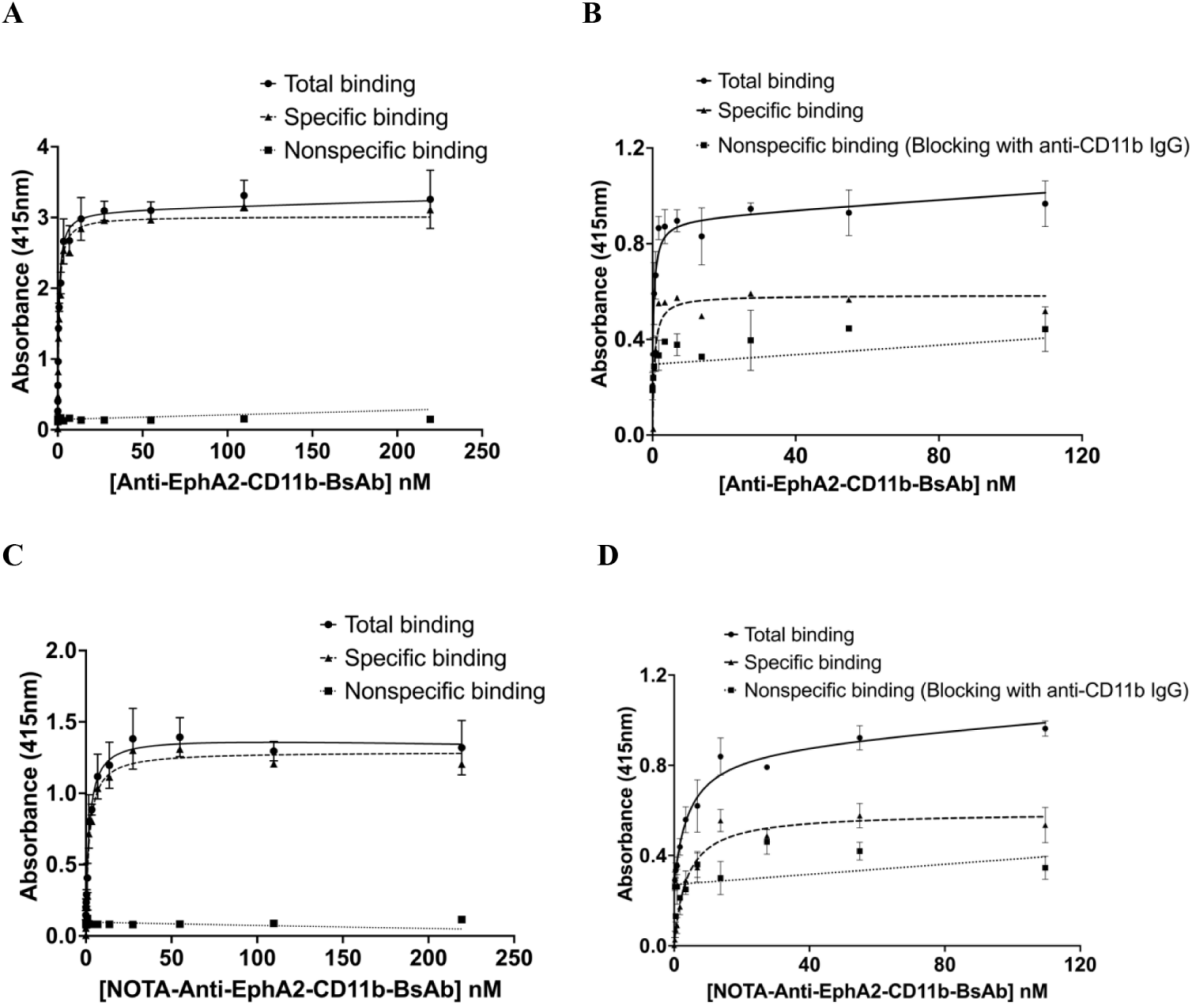
Binding of anti-EphA2-CD11b-BsAb and NOTA-anti-EphA2-CD11b-BsAb to EphA2 and CD11b antigens. (A and B) Saturation binding curves of anti-EphA2-CD11b-BsAb to immobilized human EphA2 antigen and CD11b receptor protein on the cell surface of Raw 264.7 cells, respectively. (C and D) ELISA and cell-ELISA showing the binding of NOTA-anti-EphA2-CD11b-BsAb to EphA2 and CD11b, respectively

### Chelator Conjugation, Radiochemical Yield, and Purity of Copper-64 Radiolabeling Reaction

The molar ratios of NOTA to anti-EphA2-CD11b-BsAb ranged between 1.8 and 4.5 (*n* = 6), as calculated using the UV280, UV245 formula by Hamblett et al.^30^ Conjugation to NOTA facilitated successful radiolabeling of anti-EphA2-CD11b-BsAb with ^64^Cu, with a high resultant radiochemical yield and purity of 95 to 100%. Representative radio-iTLC (Figure 3A) and radio-SEC-HPLC (Figure 3B) chromatograms demonstrate that both media can resolve unchelated ^64^Cu or any remaining NOTA that was not removed during purification.

**Figure 3:**
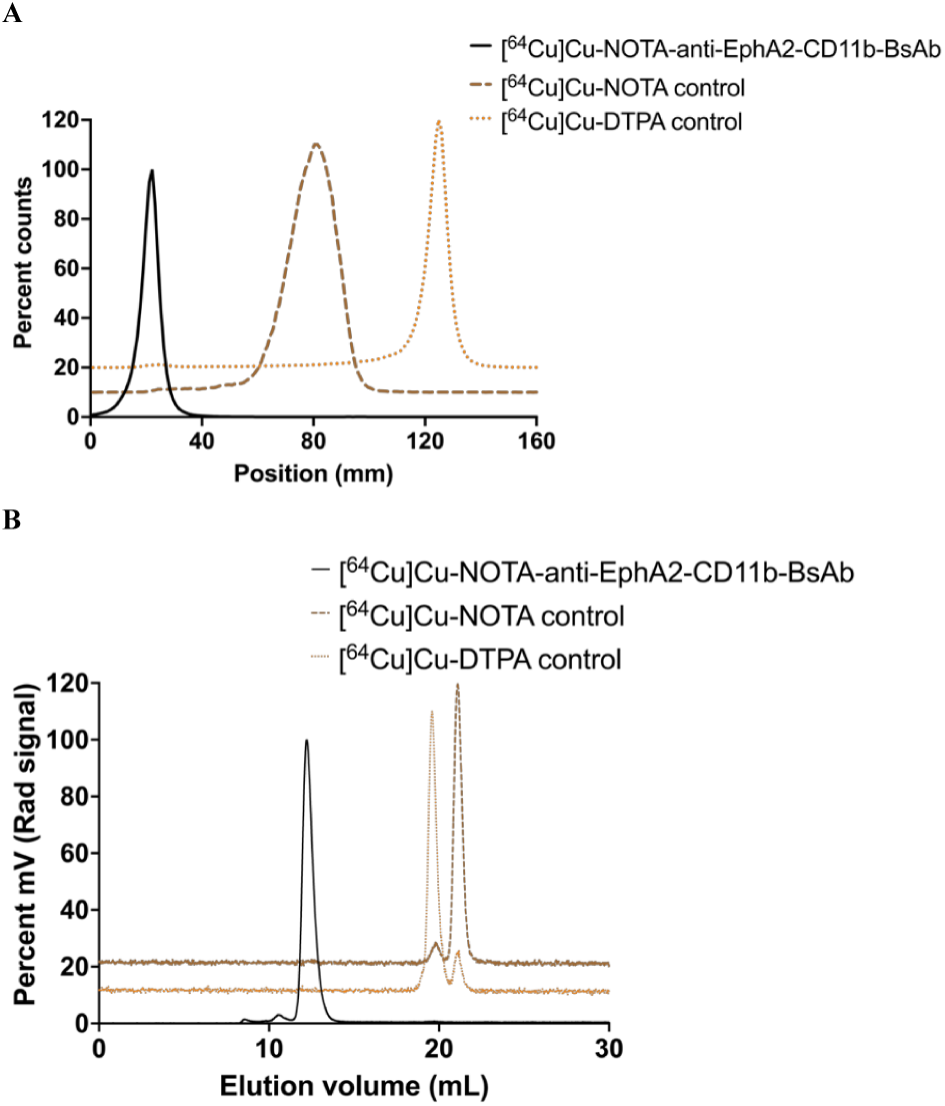
Radiochemical purity of [^64^Cu]­Cu-NOTA-anti-EphA2-CD11b-BsAb. (A) Radio-iTLC and (B) radio-SEC-HPLC chromatograms of [^64^Cu]­Cu-NOTA-anti-EphA2-CD11b-BsAb show high radiochemical purity

### Immunoreactivity of [^64^Cu]­Cu-NOTA-anti-EphA2-CD11b-BsAb

Following conjugation and radiolabeling, anti-EphA2-CD11b-BsAb retained its biological activity, binding effectively to molecular targets. Using streptavidin-coated magnetic beads and the human EphA2 ectodomain, a percent immunoreactivity of 87.30 ± 4.62% (*n* = 3) was obtained. Minimal nonspecific binding (3.29 ± 3.06%) was observed, as shown by the no-antigen control (Figure 4A).

**Figure 4:**
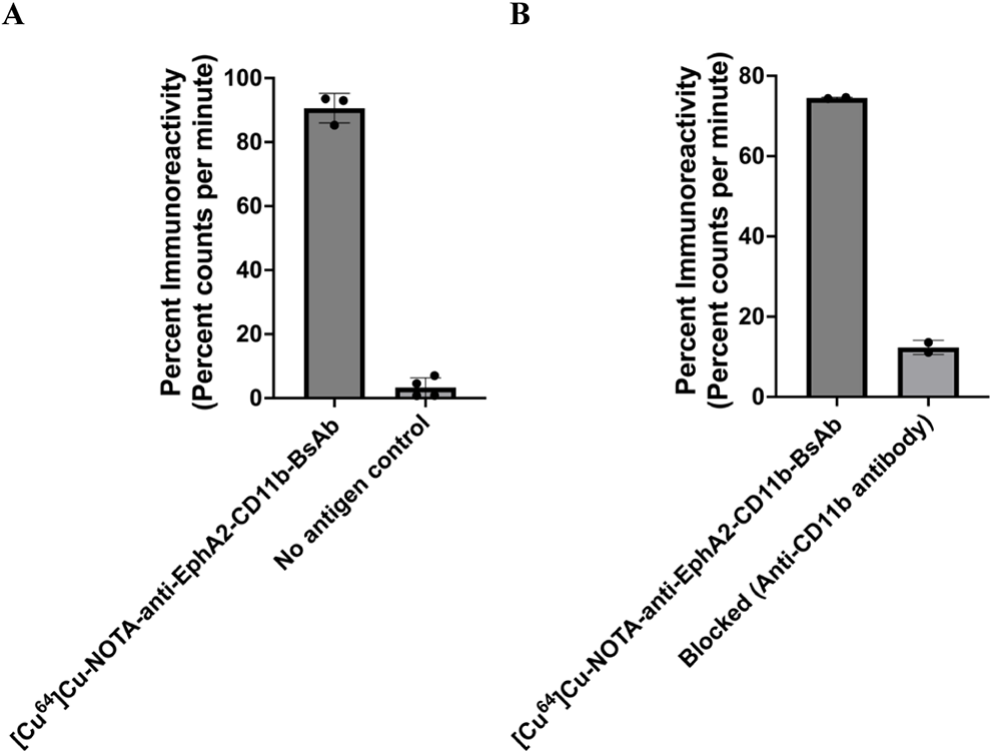
Immunoreactivity of [^64^Cu]­Cu-NOTA-anti-EphA2-CD11b-BsAb. (A) The percent bindable fraction of [^64^Cu]­Cu-NOTA-anti-EphA2-CD11b-BsAb to the human EphA2 antigen, as determined using the magnetic bead method, showed that >80% of anti-EphA2-CD11b-BsAb retained the ability to bind to human EphA2. (B) High percentage immunoreactivity was recorded for the CD11b portion of the radioimmunoconjugate using RAW 264.7 cells. The specificity of binding was determined via competitive inhibition with anti-CD11b-IgG (clone M1/70)

For the CD11b portion of the radioimmunoconjugate, immunoreactivity of 74.48 ± 0.19% (*n* = 2) was obtained using RAW 264.7 cells, which was blocked to 12.34 ± 1.75% by anti-CD11b-IgG, indicating specificity of binding (Figure 4B).

### Stability of [^64^Cu]­Cu-NOTA-anti-EphA2-CD11b-BsAb in Mouse Serum and PBS

The stability of [^64^Cu]­Cu-NOTA-anti-EphA2-CD11b-BsAb in mouse serum at 37 °C and PBS at ambient temperature, as determined using radio-iTLC, showed that neither ^64^Cu nor [^64^Cu]­Cu-NOTA dissociated from the complex over time (Figure 5A,B, respectively). To assess the integrity of anti-EphA2-CD11b-BsAb over time, the stability of the radioimmunoconjugate in mouse serum at 37 °C was additionally evaluated via radio-SEC-HPLC (Figure 5C). Analysis of peak areas indicated >80% stability at 24 h and >70% at 48 h postincubation (Figure 5D).

**Figure 5:**
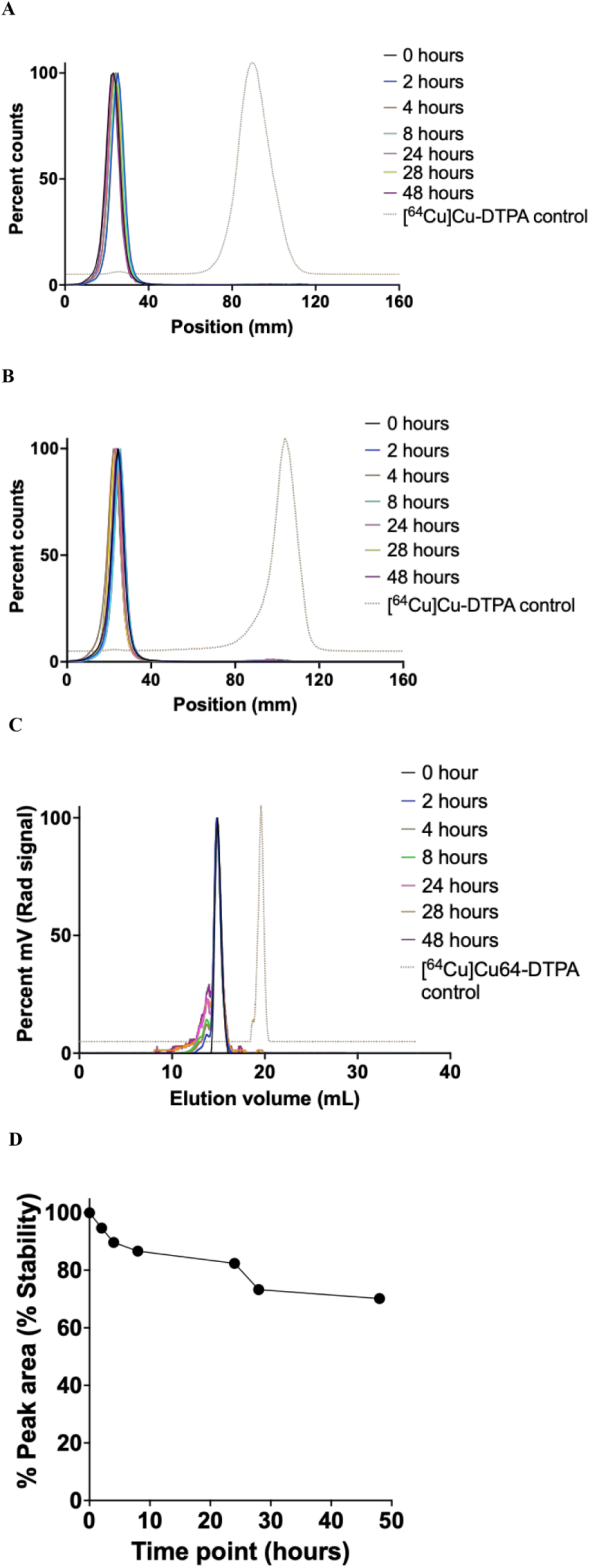
In vitro stability of [^64^Cu]­Cu-NOTA-anti-EphA2-CD11b-BsAb in PBS and mouse serum. (A and B) Radio-iTLC chromatograms showing the stability of [^64^Cu]­Cu-NOTA-anti-EphA2-CD11b-BsAb in mouse serum and PBS, measured at various time points postincubation at 37 and 27 °C, respectively. (C) Radio-SEC-HPLC chromatograms showing the stability of [^64^Cu]­Cu-NOTA-anti-EphA2-CD11b-BsAb in 50% mouse serum over time, specifically the intactness of the BsAb. (D) Analysis of peak areas of radio-SEC-HPLC chromatograms of [^64^Cu]­Cu-NOTA-anti-EphA2-CD11b-BsAb at various time points postincubation in mouse serum

### Tumor Targeting and Pharmacokinetic Distribution of [^64^Cu]­Cu-NOTA-anti-EphA2-CD11b-BsAb in HT1080-Fibrosarcoma—Bearing Athymic Nude Mice

To determine the tumor-targeting and pharmacokinetics of [^64^Cu]­Cu-NOTA-anti-EphA2-CD11b-BsAb, ex vivo biodistribution analyses and in vivo PET/CT imaging were performed using athymic nude mice with HT1080-fibrosarcoma xenografts. The radiolabeled conjugate exhibited rapid uptake in HT1080 xenografts, with 5.35 ± 2.24%ID/g, 4.44 ± 1.90%ID/g, and 4.10 ± 0.60%ID/g at 4, 24, and 48 h postinjection (p.i.), respectively (*n* = 4). Additionally, due to the lack of an intact Fc domain, there was fast blood clearance of the BsAb, with tumor-to-blood ratios of 0.87 at 4 h and 20 at both 24 and 48 h p.i.

Aside from the tumor, liver, spleen, and bone, minimal uptake of the radioimmunoconjugate was observed in other organs (Figure 6A), indicating that anti-EphA2-CD11-BsAb is a promising targeting molecule candidate for cancer therapeutics. The high liver uptake is attributed, in part, to hepatic clearance of the BsAb, given its molecular weight surpasses the renal excretion threshold. The spleen and bone (marrow), in addition to the liver, serve as CD11b antigen sinks, meaning they contain high levels of CD11b-expressing cells.^26,28,31^ Consequently, these organs recorded high uptake driven by the CD11b arm of the BsAb. This could potentially hinder the effective therapeutic application of this BsAb, as the spleen and bone would be vulnerable to undesirable side effects from cytotoxins the BsAb is employed to deliver, like radionuclides.^32,33^ However, there is a positive aspect: administering nonradiolabeled BsAb and anti-CD11b-IgG together with the dose occupied these antigen sinks, significantly reducing the uptake of [^64^Cu]­Cu-NOTA-anti-EphA2-CD11b-BsAb in these organs (*p* = 0.0104 and 0.0040 for spleen and bone, respectively), resulting in a concurrent increase in overall tumor uptake compared to mice receiving the dose alone without blocking (*p* = 0.0175). This phenomenon was also observed in the lungs (*p* = 0.0078), which are immune cell reservoirs and thus CD11b-rich^27^ (Figure 6B). This blocking effect and reduction in uptake were not seen in other tissues, which demonstrates CD11b specificity.

**Figure 6:**
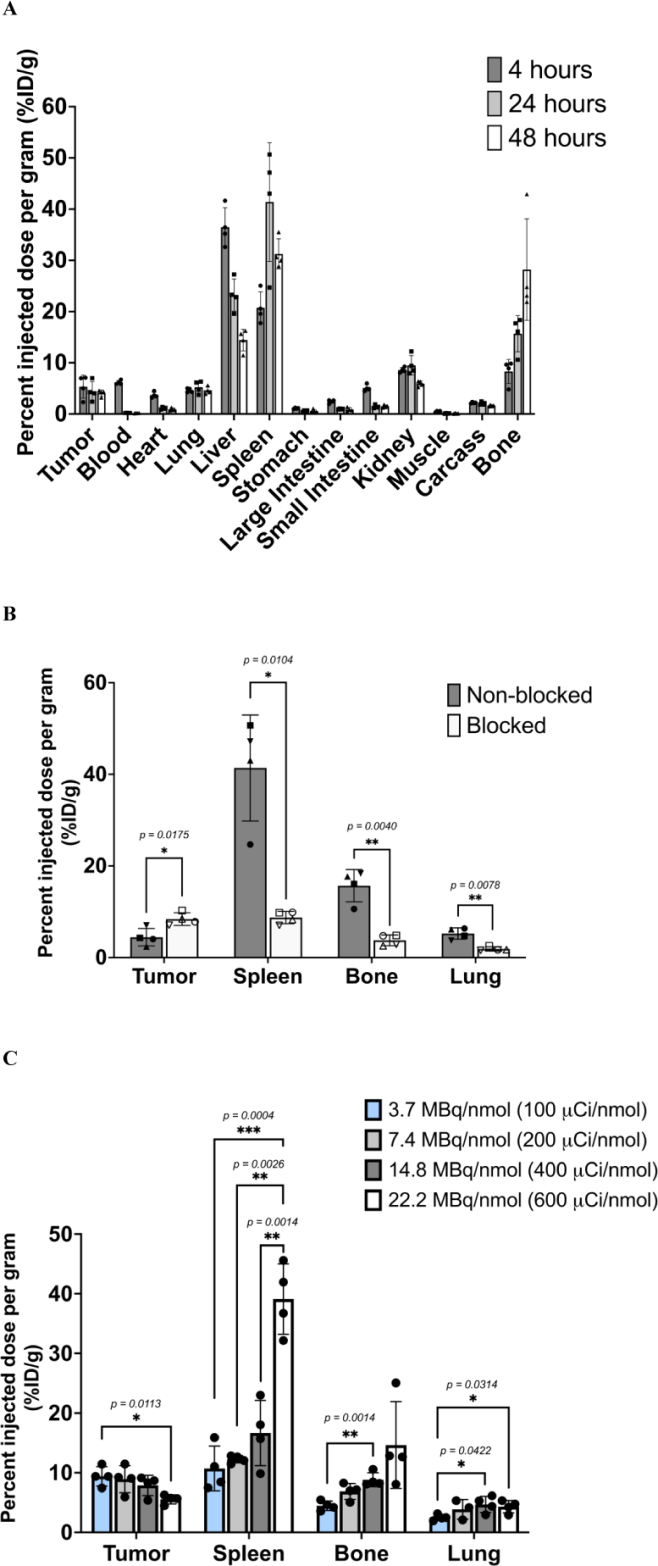
Ex vivo biodistribution analyses of [^64^Cu]­Cu-NOTA-anti-EphA2-CD11b-BsAb in HT1080- fibrosarcoma-bearing mice and molar activity optimization studies. (A) Tumor uptake and pharmacokinetic localization of [^64^Cu]­Cu-NOTA-anti-EphA2-CD11b-BsAb were determined by ex vivo biodistribution analyses of HT1080-fibrosarcoma-bearing athymic nude mice injected with a 0.26 MBq (7 μCi) dose of the radioimmunoconjugate. (B) Ex vivo biodistribution analysis of [^64^Cu]­Cu-NOTA-anti-EphA2-CD11b-BsAb at 24 h p.i., depicting significant changes in spleen, bone, lung, and tumor uptake upon coadministration of the dose with nonradiolabeled anti-EphA2-CD11b-BsAb and anti-CD11b-IgG, compared to nonblocked mice (*n* = 4). (C) Additional dose optimization studies show the enhanced tumor uptake and reduced accumulation of [^64^Cu]­Cu-NOTA-anti-EphA2-CD11b-BsAb in CD11b-rich organs at lower molar activities relative to higher molar activities

To further investigate the utility of nonradiolabeled BsAb for competitive inhibition and reduction of uptake in CD11b antigen sinks, with a concurrent increase in tumor uptake, different molar activities were assessed. At lower molar activities, with higher amounts of nonradiolabeled BsAb relative to higher molar activities, uptake in the spleen, bone, and lungs was significantly reduced. Tumor uptake at 3.7 MBq/nmol (100 μCi/nmol) was significantly higher compared to tumor uptake at 22.2 MBq/nmol (600 μCi/nmol) (Figure 6C).

Analysis of the in vivo PET images further demonstrates the tumor-targeting properties of [^64^Cu]­Cu-NOTA-anti-EphA2-CD11b-BsAb, with Standardized Uptake Values (SUV_mean_) of 0.77 ± 0.28 and 0.84 ± 0.31 at 24 and 48 h p.i., respectively (*n* = 2) (Figure 7).

**Figure 7:**
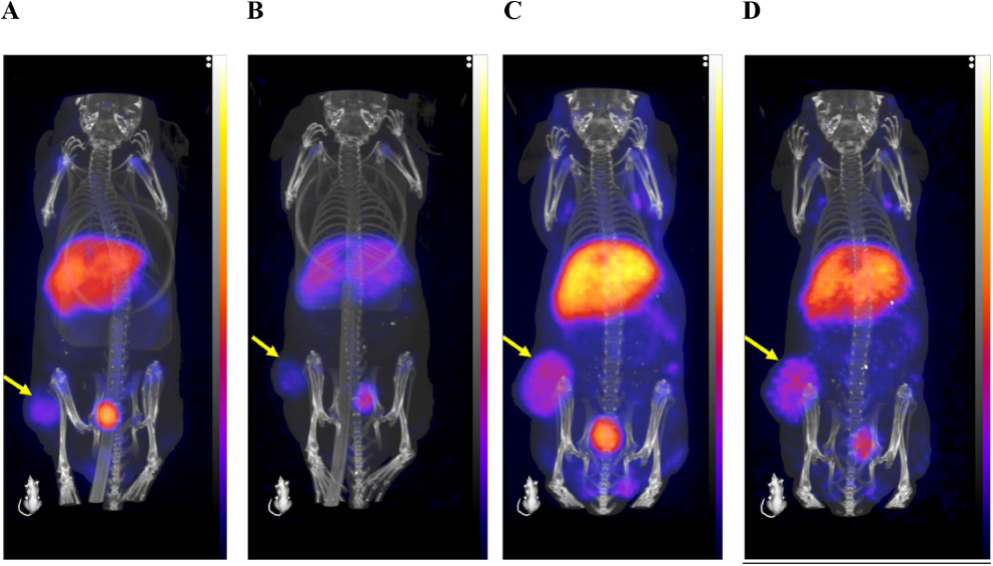
Tumor targeting and pharmacokinetics of [^64^Cu]­Cu-NOTA-anti-EphA2-CD11b-BsAb. (A and B) Representative PET/CT images at 24 and 48 h p.i., respectively, show the in vivo tumor-targeting and pharmacokinetics of [^64^Cu]­Cu-NOTA-anti-EphA2-CD11b-BsAb in HT1080- fibrosarcoma-bearing athymic nude mice. The mice received an 11.1 MBq (300 μCi) dose. (C and D) Representative 24 and 48 h p.i. PET/CT images of mice receiving a coadministration of the dose and nonradiolabeled BsAb and anti-CD11b-IgG

## Discussion

The essential new knowledge this study provides is that anti-EphA2-CD11b-BsAb—a dual-targeting molecule for both EphA2, a tumor-associated antigen, and CD11b, a cell-surface marker of TAMCs—demonstrated receptor-mediated tumor-targeting abilities, although substantial uptake is seen in CD11b-expressing organs. However, its pharmacokinetics and potential theranostic applications are significantly enhanced by occupying and inhibiting uptake in CD11b antigen sink organs, demonstrating the influence of the antigen sink on tracer pharmacokinetics. This conclusion is based on the following experimental evidence. First, recombinant anti-EphA2-CD11b-BsAb, with a sequence design comprising two antibody fragments—an anti-EphA2 minibody and an anti-CD11b single-domain antibody—was found to be stable, pure, and, most importantly, capable of binding to both molecular targets with high affinities. Next, when radiolabeled with ^64^Cu after conjugation to a bifunctional chelator, [^64^Cu]­Cu-NOTA-anti-EphA2-CD11b-BsAb exhibited high immunoreactivity and good in vitro stability. Then, PET/CT imaging and ex vivo biodistribution showed receptor-mediated tumor accumulation of [^64^Cu]­Cu-NOTA-anti-EphA2-CD11b-BsAb, along with uptake in CD11b antigen-rich organs. However, administration of nonradiolabeled anti-CD11b-IgG and BsAb, along with molar activity optimization mitigated the high uptake in the spleen, bone, and lung, concurrently increasing tumor uptake, thus addressing the influence of CD11b-positive tissues on tumor accumulation.

Due to its high expression on tumor cells and markedly low expression in healthy tissues, EphA2 is an attractive molecular target, and considerable efforts have been made to target EphA2 for various cancer therapeutic applications. Some studies have utilized agonistic and antagonistic anti-EphA2 monoclonal antibodies, such as EA2 and DS-8895a, respectively, for in vivo tumor growth inhibition.^34,35^ Other studies have developed anti-EphA2 antibody/peptide-drug conjugates to facilitate the targeted delivery of cytotoxins to tumor sites.^36–38^ Therefore, developing targeting molecules that bind to either EphA2 alone or EphA2 and other markers simultaneously could maximize therapeutic efficacy.

Another marker that would be suitable for BsAb targeting is CD11b, which is expressed on TAMCs. The immunosuppressive and tumor growth-promoting effects of TAMCs have been mitigated by targeting the cell surface protein CD11b with antibodies and small-molecule agonists.^18,20–23^ The development of CD11b-targeted antibody-drug conjugates for therapy has also been explored, with examples like the anti-CD11b-CpG conjugate.^19^ Outside of therapeutic applications, CD11b has been targeted for the noninvasive visualization of tumors in vivo. Examples include the use of zirconium-89-anti-CD11b-IgG and fluorine-18-anti-CD11b-VHH for PET imaging of glioblastoma and melanoma tumors, respectively.^24,25^ Based on the promising outcomes seen from singly targeting EphA2 and CD11b, simultaneous targeting with a bispecific molecule, such as the anti-EphA2-CD11b antibody used in this study, could enhance overall therapeutic effectiveness.

Dual-targeting molecules play a vital role in cancer therapeutics. Like monotargeting antibodies and peptides, bispecific antibodies, and peptides are typically employed to bind to and either neutralize or activate their targets or deliver a therapeutically active cytotoxic agent.^39,40^ By targeting two tumor-associated antigens, bispecific antibody-drug conjugates like anti-HER2/CD63, anti-HER2/PRLR, and anti-EGFR/HER3 antibodies have been shown to improve the delivery of toxins to tumors.^41–43^ For targeted radionuclide therapy, a lutetium-177 (^177^Lu)-labeled bispecific antibody targeting EGFR and c-MET had high uptake in a nonsmall cell lung cancer tumor model.^44^ A likely therapeutic application of the bispecific antibody designed in this study would be the delivery of cytotoxic molecules, such as toxins or radiometals, to tumor sites.

Due to the high uptake in CD11b-rich organs like the spleen and bone marrow, the therapeutic application of anti-EphA2-CD11b-BsAb for delivering cytotoxic payloads is limited by potential damage to these organs. Because CD11b is expressed across a wide range of immune cells within and outside the tumor microenvironment, including macrophages, monocytes, neutrophils, eosinophils, and basophils, there is a risk of undesirable side effects like myelotoxicity and neutropenia. As noted in the results, the potential pitfall of high uptake in CD11b-rich tissues was mitigated by varying the molar activity or preloading, although further optimizations are required.

In the approach of preloading, the unlabeled or nontoxic ligand is preadministered to competitively inhibit targeting antibody accumulation in on-target, off-tissue sites. This has been utilized in other studies involving mice and human patients.^45–47^ For example, preloading reduced on-target, off-tissue uptake of ^89^Zr-rituximab,^46,47^ ^89^Zr-trastuzumab,^48^ and ^89^Zr-5B1,^45^ thus maximizing their uptakes in tumors. For the effective use of this preloading approach and the future therapeutic application of an anti-EphA2-CD11b-BsAb armed with a toxic moiety, identifying the optimal amount of the nontoxic antibody and the timing of its administration in relation to the cytotoxic dose would be an important consideration. This precision would be essential for achieving maximum tumor uptake while minimizing any negative impact on nontarget antigen sink tissues.

While accumulation in on-target, off-tissue sites was mitigated through changes in molar activity, a limitation of this study is the challenge of deciphering the specific contributions of CD11b and EphA2 to tumor uptake. Other radiotracers comprising monospecific antibodies targeting either EphA2 or CD11b have exhibited antigen-specific accumulation in tumors, with minimal uptake in nontarget tissues for the former and substantial uptake in antigen sink organs for the latter.^24,49^ For the BsAb used in this study, the results support that uptake in tissues such as bone and spleen is driven by CD11b binding, but we are unable to clearly delineate the roles of EphA2 and CD11b in the overall tumor uptake and pharmacokinetics of the BsAb.

In conclusion, this study introduces a novel targeting molecule, Anti-EphA2-CD11b-BsAb, showing potential for cancer therapeutic applications. Given the near-ubiquitous abundance of EphA2 and CD11b in tumors, this BsAb could be utilized across diverse cancer types as a therapeutic or theranostic.

## Experimental Procedures

### Anti-EphA2-CD11b Bispecific Antibody Sequence Design, Production, and Purification

The anti-EphA2-CD11b-BsAb sequence was engineered from two antibody fragments: an anti-EphA2 minibody comprised of V_H_, V_L_,^29^ and CH_3_ domains, and an anti-CD11b single-domain antibody (VHH)^25^ (Figure 1A). A histidine tag was placed at the C-terminus of the construct. The anti-EphA2-CD11b-BsAb was recombinantly produced in Chinese Hamster Ovary (CHO) cells and purified via immobilized metal affinity chromatography (Nickel-NTA).

### Assessment of Purity, Stability, and Molecular Weight of Recombinant Anti-EphA2-CD11b-BsAb

SDS-PAGE was performed to assess the purity and molecular weight of the recombinant anti-EphA2-CD11b-BsAb. Nonreduced and reduced BsAb were loaded onto a 10% polyacrylamide gel, run at 200 V for 30 min, and then stained with Coomassie blue. The gel was destained with a solution of 15% acetic acid and 25% methanol before imaging. To further confirm molecular weight, assess purity, and detect aggregation, SEC-HPLC was performed using a Superdex 200 Increase 10/300 GL column with PBS at a flow rate of 1 mL/min. Protein standards of varying molecular weights were also run under the same conditions.

### Saturation Binding Assay

Enzyme-linked immunosorbent assays (ELISA) were conducted to determine the binding affinities of anti-EphA2-CD11b-BsAb to the target antigens EphA2 and CD11b. EphA2 ectodomain (Biorbyt, Cambridge, UK) was immobilized onto a 96-well microtiter plate (0.1 μg per well) and incubated for 2 h. Following incubation, the wells were blocked with Tris-buffered saline (TBS), 1% bovine serum albumin (BSA), and 0.05% Tween-20 detergent for 30 min. After washing with TBS containing 0.1% BSA and 0.05% Tween-20 detergent, varying concentrations of anti-EphA2-CD11b-BsAb, ranging from 110 nM to 7 pM, were added (100 μL). Following 2 h of incubation and washing, 0.1 μg of secondary antibody, anti-His-HRP (100 μL), was added to each well. After an additional hour of incubation, the wells were washed, and HRP substrate was added. The HRP–HRP substrate reaction was terminated after 15 min with 2% oxalic acid, and absorbance was measured at 415 nm.

Binding to CD11b was determined using RAW 264.7, a CD11b-expressing murine macrophage cell line. Cells were seeded overnight in a 96-well microtiter plate at a density of 100,000 cells per well. After gentle washing with ice-cold PBS containing 0.5% BSA, different concentrations of the BsAb, prepared via serial dilutions with ice-cold PBS containing 1% BSA, were added to the respective wells and incubated for 1 h at 4 °C with shaking. All incubations were conducted at this temperature unless otherwise specified. For blocking to assess specificity, 100 nM (3 μg) of anti-CD11b-IgG (Clone M1/70) was added to the cells, incubated for an hour, and washed prior to the addition of the BsAb. The cells were subsequently washed and incubated for 1 h with the secondary antibody, anti-His-HRP. Next, HRP substrate was added, and after 15 min of incubating the reaction at room temperature, the absorbance was read at 415 nm. The data obtained for both binding assays were fitted to a curve using the nonlinear regression option in GraphPad Prism 10. To generate specific binding curves, nonspecific binding was linearized (for CD11b) and then subtracted from total binding.

### Conjugation of Anti-EphA2-CD11b-BsAb to a Bifunctional Chelator NOTA-SCN

A 20-fold molar equivalent of NOTA-SCN, solubilized in DMSO at a stock concentration of 25 mg/mL (44.65 mM), was added to 1–2 mg of anti-EphA2-CD11b-BsAb in PBS. The pH of the mixture was adjusted to 8.5 using 0.1 M Na_2_CO_3_, and the reaction was incubated at room temperature for 2.5 h. Excess NOTA-SCN was subsequently removed via gel filtration using the Zeba spin desalting column, 7K MWCO (Fisher Scientific, Hanover Park, IL, USA), with simultaneous buffer exchange to PBS, pH 7.5. The molar ratio of NOTA to BsAb was calculated using the formula described by Hamblett et al., based on absorbance values of the NOTA-anti-EphA2-CD11b-BsAb conjugate at UV280 and UV245.^30^ Extinction coefficients of NOTA at UV280 and UV245 were calculated via plots of molar concentration against absorbance.

### Radiolabeling of NOTA-Anti-EphA2-CD11b-BsAb Conjugate

Following chelator conjugation, NOTA-Anti-EphA2-CD11b-BsAb was radiolabeled with ^64^Cu at a molar activity of 14.8 MBq/nmol (400 μCi/nmol). The conjugate was first buffer-exchanged from PBS to radiolabeling buffer, 0.1 M ammonium acetate, using the Zeba Desalting Spin Column, 7K MWCO. The desired activity of ^64^Cu (CuCl_2_) was aliquoted from the stock and diluted to a final volume of 50 μL with radiolabeling buffer. This was added to the conjugate, mixed, the pH adjusted to 6.0 using 0.5 M HCl, and then incubated at room temperature for an hour. Following incubation, radiochemical yield and purity were determined through radio-iTLC and radio-SEC-HPLC. For radio-iTLC, silica gel-coated TLC paper and 50 mM sodium citrate buffer were used as the stationary and mobile phases, respectively. A 5 μL aliquot of the radiolabeling reaction mix was added to 5 μL of 50 mM DTPA to chelate free ^64^Cu, and then 2 μL was applied to the TLC paper. For radio-SEC-HPLC, the Superdex 200 Increase 10/300 GL column was used with PBS as the elution buffer. DTPA was added to an aliquot of the reaction mixture before sample application.

### Immunoreactivity Assay

To quantify the bindable fraction of Anti-EphA2-CD11b-BsAb to human EphA2 following chelator conjugation and radiolabeling, a magnetic bead-based immunoreactivity assay was performed.^50^ About 18.5–37 kBq (0.5–1 μCi) of [^64^Cu]­Cu-NOTA-anti-EphA2-CD11b-BsAb was added to a 100-fold molar excess of biotinylated human EphA2 antigen, which had been previously incubated for 2 h with streptavidin-coated magnetic beads. After 2 h of incubation, the beads were washed thrice, and the activity was measured using a gamma counter, alongside the supernatants from the washes. The percent immunoreactivity of [^64^Cu]­Cu-NOTA-anti-EphA2-CD11b-BsAb for the EphA2 antigen was then calculated, factoring out nonspecific binding, which was determined by no-antigen controls.

For CD11b, 9.25 kBq (0.25 μCi) of [^64^Cu]­Cu-NOTA-anti-EphA2-CD11b-BsAb was added to 2 × 10^7^ RAW 264.7 cells resuspended in PBS and incubated on ice for 2 h. For blocking, the cells were incubated with anti-CD11b-IgG (clone M1/70) for 1 h prior to the addition of the radioimmunoconjugate. The cells were then centrifuged, and the activities of the pelleted cells and supernatant were measured on a gamma counter to calculate the percent immunoreactivity.

### Serum and PBS Stability Tests

The in vitro stability of [^64^Cu]­Cu-NOTA-anti-EphA2-CD11b-BsAb in mouse serum and PBS was evaluated to ensure the integrity of the BsAb over time. For serum stability assessment, a 3.7 MBq (100 μCi) aliquot of the radioimmunoconjugate in 100 μL PBS was added to an equal volume of mouse serum, making a 50% serum mixture. This was incubated at 37 °C. For PBS stability, 18.5 MBq (500 μCi) of [^64^Cu]­Cu-NOTA-anti-EphA2-CD11b-BsAb in PBS was incubated at ambient temperature. At various time points postincubation, radio-iTLC and radio-SEC-HPLC analyses were performed, and radiochemical purity and peak areas of the resultant chromatograms were determined as a measure of stability over time.

### Tumor Cell Culture and Inoculation in Mice

The human fibrosarcoma model, HT1080, was employed for the assessment of tumor targeting and biodistribution of anti-EphA2-CD11b-BsAb in mice. HT1080 cells were cultured in Eagle’s Minimum Essential Medium (EMEM) supplemented with 10% fetal bovine serum (FBS). The cells were harvested, washed, resuspended in a 2:1 PBS:matrigel mixture, and then inoculated into the right flanks of female athymic nude mice at a density of 10^6^ cells per mouse. Tumors were well established, and mice were ready for subsequent analyses 21 days postinoculation. All animal experiments were conducted in accordance with guidelines and protocols approved by the University of Missouri Animal Care and Use Committee (ACUC).

### Ex Vivo Biodistribution Analysis and Dose Optimization

To determine the pharmacokinetics of [^64^Cu]­Cu-NOTA-anti-EphA2-CD11b-BsAb in HT1080- fibrosarcoma-bearing mice, 0.26 MBq (7 μCi) of the radiolabeled conjugate was administered via tail vein injection to three groups of mice (*n* = 4). At 4, 24, and 48 h p.i., these mice were euthanized, and their tumors and other organs were harvested, weighed, and their activities measured using a gamma counter for %ID/g calculations.

To demonstrate CD11b-mediated uptake in the tumor and CD11b-rich tissues, a fourth group of mice (*n* = 4) received the same dose, coadministered with anti-EphA2-CD11b-BsAb and anti-CD11b-IgG (700 μg of each) for competitive inhibition of tracer binding to the targeted receptor. Ex vivo biodistribution analysis was performed at 24 h p.i.

Further optimization to improve tumor targeting and pharmacokinetics was achieved by varying the molar activities of [^64^Cu]­Cu-NOTA-anti-EphA2-CD11b-BsAb. Keeping the amount of BsAb the same, the amount of radioactivity was varied such that, at lower molar activities, there was putatively more nonradiolabeled or “free” BsAb than at higher molar activities. The molar activities investigated were 22.2 MBq/nmol (600 μCi/nmol), 14.8 MBq/nmol (400 μCi/nmol), 7.4 MBq/nmol (200 μCi/nmol), and 3.7 MBq/nmol (100 μCi/nmol). These were administered to tumor-bearing mice at doses of 12 μCi, 8 μCi, 4 μCi, and 2 μCi, respectively, for ex vivo biodistribution analysis at 24 h p.i.

### In Vivo PET Imaging

Two groups of HT1080-fibrosarcoma-bearing mice (*n* = 2) received 11.1 MBq (300 μCi) of [^64^Cu]­Cu-NOTA-anti-EphA2-CD11b-BsAb per mouse via tail-vein injection (14.8 MBq/nmol). For one group, designated as the blocked group, the dose was coadministered with nonradiolabeled anti-EphA2-CD11b-BsAb and anti-CD11b-IgG. PET/CT images were acquired at 24 and 48 h p.i. Image analysis and SUV_mean_ quantifications were performed using VivoQuant software.

#### Safety Statement (Caution)

*The radioactive metal used in this study,* ^*64*^ *Cu is hazardous and must, therefore, be handled with caution to prevent exposure and/or contamination. Radiolabeling and handling of radiolabeled materials must be conducted by trained personnel using appropriate lead shielding and personal protective equipment (PPE).*

### Statistical Analysis

Data analyses were conducted using Prism 10 of the GraphPad software. For statistical analysis and comparison between blocked and nonblocked mice groups, Student’s *t*-test was used. Statistics were considered significant for *p* < 0.05. Error values are reported as the standard error of the mean (SEM), except for animal experiments and CD11b immunoreactivity error values, which are stated as the standard deviation (SD).

## Supplementary Material







## Notes

Anti-EphA2-CD11b-BsAb amino acid sequence and domains; binding affinities of anti-EphA2-CD11b-BsAb and NOTA-anti-EphA2-CD11b-BsAb to mouse EphA2 antigen (PDF)
(PDF)
(PDF)

## Abbreviations

BsAb: Bispecific antibody
NOTA-SCN: 2-S-(4-Isothiocyanatobenzyl)-1,4,7-triazacyclononane-1,4,7-triacetic acid
SEC: Size Exclusion Chromatography
HPLC: High-Performance Liquid Chromatography
iTLC: Instant Thin Layer Chromatography
PET: Positron Emission Tomography
CT: Computerized Tomography
IgG: Antibody
