# Supplementary material for: Developing and Characterizing the Tumor-Targeting Efficiency of an Anti-EphA2-CD11b Bispecific Antibody
Source: Bioconjug Chem. 2025 May 28;36(6):1208–17. doi: 10.1021/acs.bioconjchem.5c00070 (PMC12184676; doi:10.1021/acs.bioconjchem.5c00070)

SUPPORTING INFORMATION

Peggy A. Birikorang[a,b], Dominic M. Menendez[a,b], Robert Edinger[c], Gary Kohanbash[d*], W. Barry Edwards[a,b,e*]

a. University of Missouri-Columbia, Department of Biochemistry, 503 S College Avenue, Columbia, MO, 65211, USA
b. University of Missouri-Columbia, Molecular Imaging and Theranostics Center, 1514 Research Park Drive, Columbia, MO, 65203, USA
c. University of Pittsburgh, Department of Radiation Oncology, 200 Lothrop Street, Pittsburgh, PA, 15213, USA
d. University of Pittsburgh, Department of Neurological Surgery, 503 45th Street, Pittsburgh, PA, 15201, USA
e. University of Missouri-Columbia, Department of Chemistry, 601 S College Avenue, Columbia, MO, 65211, USA

Anti-EphA2-CD11b bispecific antibody amino acid sequence and domains

The amino acid sequence and domains of anti-EphA2-CD11b bispecific antibody are shown below. This comprises of an anti-CD11b VHH or single domain antibody, fused to the C-terminal of an anti-EphA2 minibody (V_H, V_L, CH_3) via a $(GGGS)_3$ linker.

Signal sequence – V_H domain – Linker – V_L domain – Hinge region – CH_3 domain – Linker – CD11b VHH domain – His tag

MGWSCIILFLVATATGVHSMEVQLLESGGGLVQPGGSLRLSCAASGFTFSHYMMAWVR
QAPGKGLEWVSRIGPSGGPTHYADSVKGRFTISRDNSKNTLYLQMNSLRAEDTAVYYC
AGYDSGYDYVAVAGPAEYFQHWGQGTLVTVSSGSTSGGGSGGGSGGGSSDIQMTQSPS

SLSASVGDRVTITCRASQSISTWLAWYQQKPGKAPKLLIYKASNLHTGVPSRFSGSGSGT EFSLTISGLQPDDFATYYCQQYNSYSRTFGQGTKVEIKAAALEPKSCDKTHTCPPCGGGS SGGGSGGQPREPQVYTLPPSRDELTKNQVSLTCLVKGFYPSDIAVEWESNGQPENNYKT TPPVLDSDGSFFLYSKLTVDKSRWQQGNVFSCSVMHEALHNHYTQKSLSLSPGKGGGS GGGSGGGSQVQLQESGGGLVQAGGSHNLSCTASGITFSSLAMGWFRQTPGKEREFVANI MRSGSSVFYADSVRGRFTISRDNAKNTAHLQMNSLKPEDTAVYFCAATRGAWPAEYW GQGTQVTVSSGGLPETGGHHHHHH

Binding affinities of anti-EphA2-CD11b BsAb and NOTA-anti-EphA2-CD11b-BsAb to mouse EphA2 antigen

Saturation binding assays (ELISA) were performed to determine the binding affinity of the BsAb as well as the NOTA-conjugated BsAb to mouse EphA2 antigen. Resultant binding curves are shown below (**Figure S1A and Figure S1B** respectively). K_D values recorded were 0.70 ± 0.12 nM for anti-EphA2-CD11b-BsAb 0.64 ± 0.10 nM and for NOTA-anti-EphA2-CD11b-BsAb.

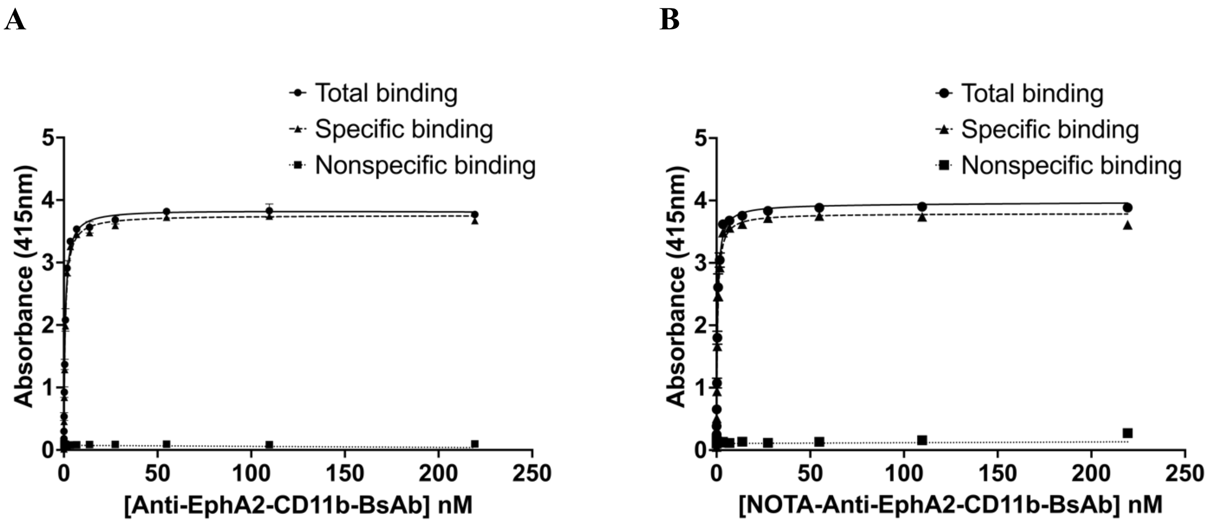

Supplement: Supplementary file 1 [file bc5c00070_si_001.pdf]
