# Supplementary figures and images for: Developing and Characterizing the Tumor-Targeting Efficiency of an Anti-EphA2-CD11b Bispecific Antibody
Source: Bioconjug Chem. 2025 May 28;36(6):1208–17. doi: 10.1021/acs.bioconjchem.5c00070 (PMC12184676; doi:10.1021/acs.bioconjchem.5c00070)

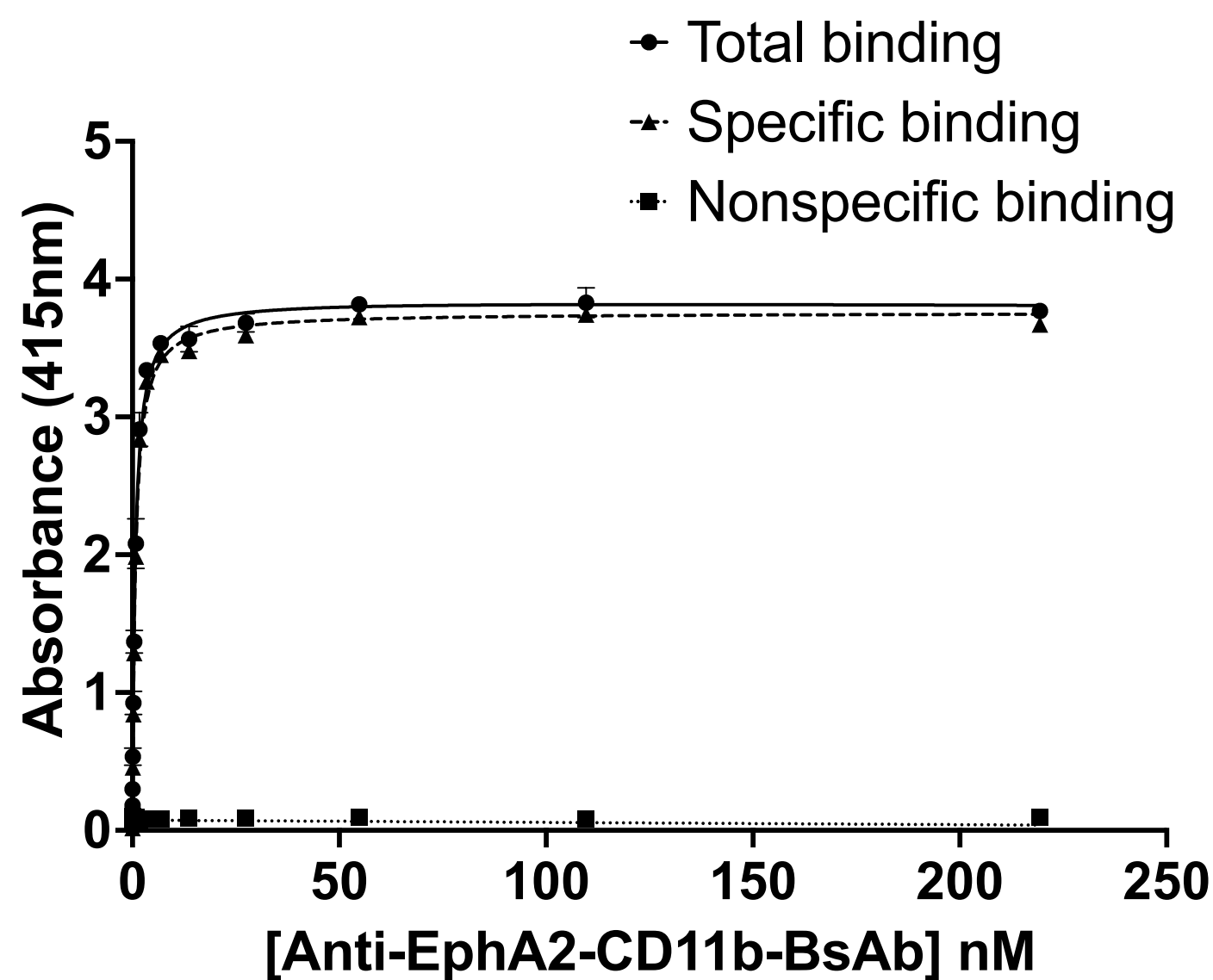

Supplement: Supplementary file 2 [file bc5c00070_si_002.pdf]

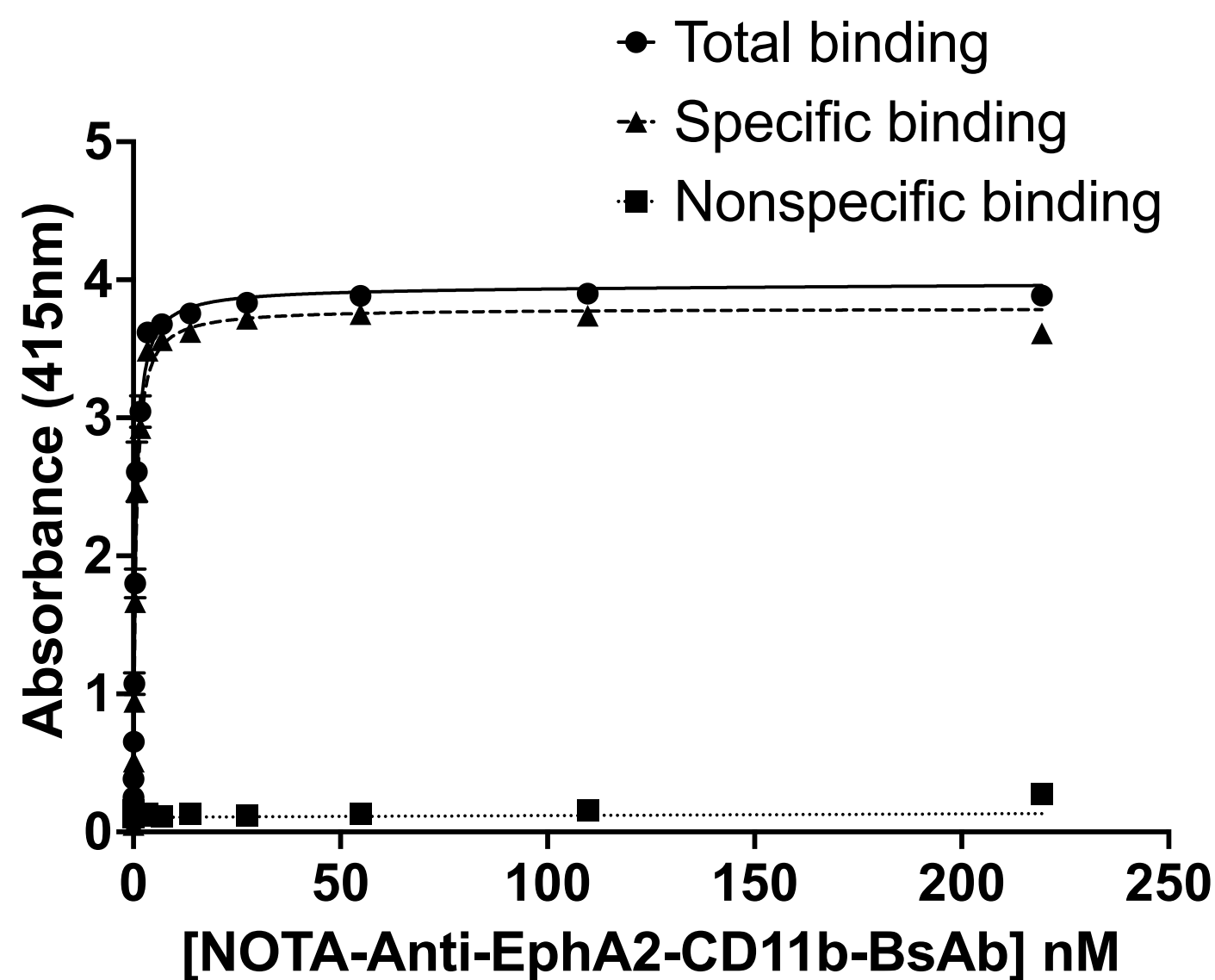

Supplement: Supplementary file 3 [file bc5c00070_si_003.pdf]
